# Subcellular Journey of Rare Cold Inducible 2 Protein in Plant Under Stressful Condition

**DOI:** 10.3389/fpls.2020.610251

**Published:** 2021-01-12

**Authors:** Hyun-Sung Kim, Won Park, Hyeon-Sook Lee, Jung-Ho Shin, Sung-Ju Ahn

**Affiliations:** ^1^Department of Bioenergy Science and Technology, Chonnam National University, Gwangju, South Korea; ^2^Bioenergy Crop Research Institute, National Institute of Crop Science, Rural Development Administration, Muan, South Korea

**Keywords:** RCI2, PMP3, NaCl stress, endocytosis, plasma membrane, protein interaction

## Abstract

Rare cold inducible 2 (RCI2) proteins are small hydrophobic membrane proteins in plants, and it has been widely reported that RCI2 expressions are dramatically induced by salt, cold, and drought stresses in many species. The RCI2 proteins have been shown to regulate plasma membrane (PM) potential and enhance abiotic stress tolerance when over-expressed in plants. RCI2 protein structures contain two transmembrane domains that are thought to be PM intrinsic proteins and have been observed at the PM and endomembranes. However, cellular trafficking of RCI2s are not fully understood. In this review, we discussed (i) general properties of RCI2s characterized in many species, (ii) the uses of RCI2s as a tracer in live cell imaging analyses and when they are fused to fluorescence proteins during investigations into vesicle trafficking, and (iii) RCI2 functionalities such as their involvement in rapid diffusion, endocytosis, and protein interactions. Consequently, the connection between physiological characteristics of RCI2s and traffic of RCI2s interacting membrane proteins might be helpful to understand role of RCI2s contributing abiotic stresses tolerance.

## Introduction

It is impossible for plants to avoid abiotic stresses because they are sessile organisms. Therefore, plants have genetically evolved toward enhanced stress tolerance against abiotic stresses through the use of complex mechanisms. The stress tolerance properties of plants vary depending on their native location and environmental conditions, and understanding the genetic and physiological mechanisms that underlie stress tolerance will improve abiotic stress tolerance in plants and help improve crop yields.

The rare cold inducible 2 (RCI2s) are putative membrane proteins that are considerably induced by environmental stresses, such as salt, cold, and drought, in most plant species, microorganisms, and nematodes, but not in higher animals (Medina et al., 2007; Kim et al., 2016; Rocha, 2016). RCI2 names, such as low temperature inducible 6 (LTI6), plasma membrane proteolipid 3 (PMP3), and sensitive to Na^+^ (SNA) can vary depending on the species. The first RCI2 to be identified was early salt stress induced 3 (ESI3) in wheatgrass under salt stress (Gulick et al., 1994). The RCI2 characteristics in *Arabidopsis* were reported by Capel et al. (1997) who investigated RCI2A and RCI2B. The unique structure of the AtRCI2 family and their subcellular localization in the plasma and inner membranes were reported by Medina et al. (2007), and these reports have been used as a basis to investigate the expression and functional analysis of RCI2s in many different species. The multifunctionality of RCI2s in various plant species was reviewed by Rocha (2016). The study concluded that RCI2 functions were associated with the mechanism involved in defense against major abiotic stresses, such as cold, drought, and salt. Ectopical expression of RCI2s enhanced abiotic stress tolerance in many species (Feng et al., 2009; Long et al., 2015; Sivankalyani et al., 2015; Yu et al., 2016; Ben Romdhane et al., 2017), but an absence of RCI2s caused sensitive responses to the stresses (Mitsuya et al., 2005, 2006). The role of RCI2s during abiotic stress defense has been well reviewed. However, the precise mechanism by which small hydrophobic RCI2s contribute to abiotic stress tolerance remains unknown.

If the detailed function of RCI2s is to be determined, then it is important to understand the unique structures of these small hydrophobic proteolipids. The RCI2s have two transmembrane domains (TMDs), and almost half the RCI2s have a hydrophilic C-terminal tail. The eight AtRCI2s in *Arabidopsis* can be divided into two types based on the presence (AtRCI2D/E/F/G) or absence (AtRCI2A/B/C/H) of the hydrophilic C-terminal tails attached to their second TMD (Medina et al., 2007). The bioenergy crop, camelina (*Camelina sativa* L), belongs to the Brassicaceae family, and the CsRCI2s in camelina are reported to be highly hydrophobic proteins compared to those in other plant species (Supplementary Figure 1). Furthermore, the CsRCI2 homologies have identities that are similar to those of AtRCI2s (Supplementary Table 1), and phylogenetic tree analyses have shown that there is a close relationship between CsRCI2s and AtRCI2s when they are grouped based on their structural similarity (Supplementary Figure 1B). The protein sequence alignment and phylogenetic tree for RCI2s clearly showed that they were divided by their structural properties (Supplementary Figure 1). Additionally, several studies have reported that the over-expression of RCI2 proteins which do not contain the C-terminal hydrophilic tail contributed physiological changes and enhance abiotic stress tolerance (Feng et al., 2009; Kim et al., 2016, 2019). However, little is known about the function of tail-type RCI2 proteins when plants are subjected to cold, drought, and salt stress.

The two TMD structure is a common property of RCI2s. In most reports, subcellular localization of RCI2s was observed in the PM and other membranes, such as the Golgi apparatus, endoplasmic reticulum (ER), and intracellular vesicles, when they were fused to fluorescence proteins (Rocha, 2016). Therefore, the structures associated with RCI2s enable them to localize in cellular membranes within the cell. However, RCI2s are small size (∼55–76 amino acids long) protein that it is not yet determined to have a transport activity contributing abiotic stress tolerance. Previous study suggested that the functions of RCI2s were not provided by its single expression but through their interaction with functional proteins related to the abiotic stresses defense mechanism (Kim et al., 2019). From this result, it was speculated that the function of RCI2 is related to protein interactions important for cellular homeostasis through vesicle trafficking, such as endocytosis.

## Changes to RCI2 Expression in Response to Abiotic Stresses

The RCI2 expressions are markedly induced by cold stress, drought, and saline stress (Medina et al., 2007; Rocha, 2016). Many reports have suggested that osmotic unbalance and water deficiency induce RCI2 expression in plant cells. The expression profiles of the *RCI2/PMP3* responses to abiotic stresses in various species have been reviewed by Rocha (2016). These include *Arabidopsis* (Capel et al., 1997; Medina et al., 2001, 2007; Nylander et al., 2001), maize (Fu et al., 2012; Zhao et al., 2014), barley (Goddard et al., 1993; Ueda et al., 2004), alfalfa and barrel medic (Long et al., 2015), wheatgrass (Gulick and Dvořák, 1992; Galvez et al., 1993), strawberry (NDong et al., 1997), *Physcomitrella patens* (Kroemer et al., 2004), sheepgrass (Inada et al., 2005), rice (Morsy et al., 2005; Kim et al., 2007; Chang-Qing et al., 2008), wheat (Imai et al., 2005), alkali grass (Chang-Qing et al., 2008), mandarin (Gimeno et al., 2009), plantain (Feng et al., 2009; Liu et al., 2012), red sage (Wang D. et al., 2013), smooth cordgrass (Baisakh et al., 2008; Baisakh and Subudhi, 2009), and *Aulacomnium turgidum* (Liu et al., 2010). Additionally, the *RCI2/PMP3* gene expression profiles have been updated for camelina (Kim et al., 2016, 2020), pearl millet (Yeshvekar et al., 2017), and cucumber (Zhou et al., 2020).

**FIGURE 1:**
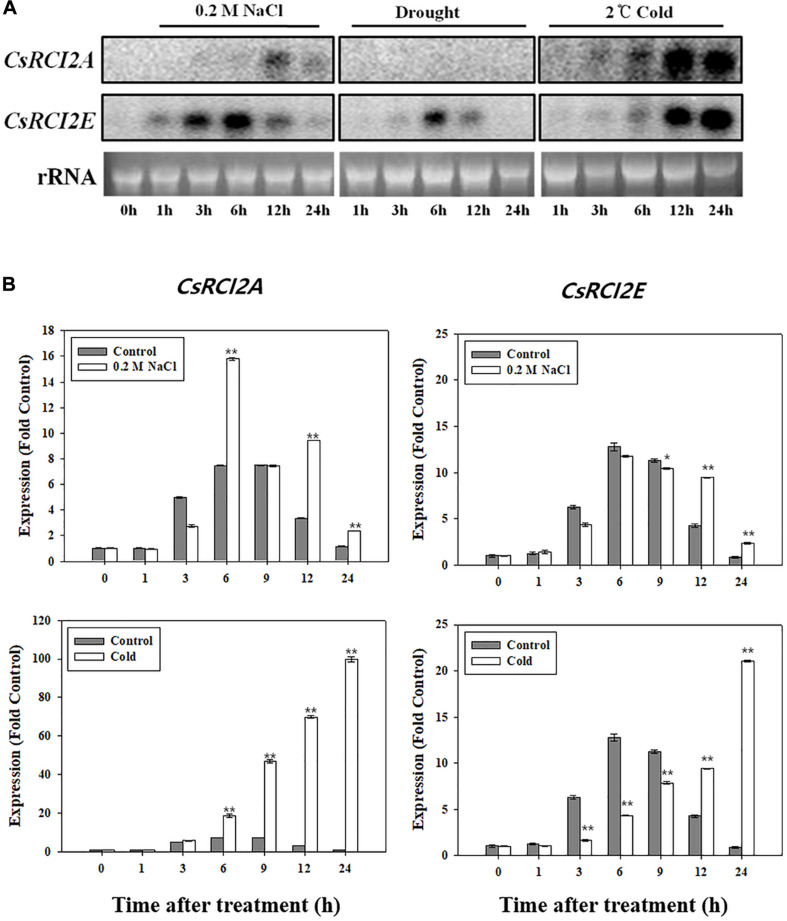
Expression changes in *CsRCI2A* and *CsRCI2E* under NaCl, cold, and mannitol stress. The expression of genes were analyzed by RNA blot **(A)** and quantitative RT-PCR analysis **(B)**. The *CsRCI2A* and *CsRCI2E* revealed typical expression of RCI2s induced by cold and NaCl stresses over time (Kim et al., 2016).

In *Arabidopsis*, *AtRCI2* expressions were considerably induced by cold, abscisic acid, dehydration, and NaCl, whereas the *AtRCI2C* and *AtRCI2H* level was not significantly different from that of the control (Medina et al., 2007). The expressions of *CsRCI2A/E* in camelina, which have high homology to *AtRCI2s* were considerably induced by cold, drought, and NaCl (Figure 1). The expressions of ten *ZmRCI2* members, which are involved in the maize response to drought stress, either increased (*ZmRCI2-1, 2, 6, 7*) or decreased (*ZmRCI2-3, 5, 8, 9, 10*) (Zhao et al., 2014). These results indicated that the expression of each *RCI2* in the plant species had individual characteristic responses to its corresponding abiotic stresses. The RCI2s may not respond to all abiotic stresses, but may react to a specific stress type depending on their promoter. Upstream region of RCI2 was reported to be regulated by C-repeat binding factor (CBF) in rice (Kim et al., 2007), but this needs to be confirmed by investigating the transcription factor that regulates the expression of RCI2s under salt stress.

Medina et al. (2007) reported that *AtRCI2s* showed different expressions depending on the tissue examined, such as leaves, stems, roots, flowers, and seeds. Additionally, modification of the *RCI2* promoter changed the localization range in xylem and phloem (Chidambaram et al., 2015). This suggested that the expression and localization of RCI2s were dependent on the promoter. At the germination stage, the expression of AtRCI2A and AtRCI2B had increased at days 1 and 2, but returned to the control level at 3 days after germination according to a GUS analysis using the native promoter for each RCI2 in *Arabidopsis* (Medina et al., 2001). Therefore, the RCI2 expressions may vary depending on the development stage and their responses to environmental stimulation. Furthermore, the regulation of each RCI2 expression may be combined with tissue specific expression and that this combination contributes to cell homeostasis under various abiotic stresses.

The gene expression of *RCI2s* has been widely reported. However, few protein accumulation analyses have been performed by western blotting. Furthermore, it is difficult to synthesize antibodies for the no-tail type RCI2s because of their hydrophobic properties. However, the accumulations of a few RCI2s, such as CsRCI2E and CsRCI2F in camelina, have been determined by analysis of the tail types (Kim et al., 2019). Both CsRCI2E and CsRCI2F accumulation at the PM was induced under NaCl stress in a time- and dose-dependent manner. The CsRCI2E accumulation was more significantly induced by NaCl concentration compared to CsRCI2F. The different RCI2 protein accumulation levels might be caused by their differing gene expression characteristics. The diverse gene and protein expression properties among RCI2 members suggest that they may have different roles in the maintenance of cell homeostasis and the plant response to various abiotic stresses.

## Properties of Plasma Membrane RCI2 Proteins

Most of the previous RCI2s studies investigated the hydrophobic two TMD structure. Many prediction results have shown that the RCI2 protein structure has two regions that are predicted to be TMDs, but several proteins have hydrophilic N- or C-terminal residues that resemble a tail (Supplementary Figure 2). The properties of RCI2/PMP3 have classified it as a proteolipid. Rocha (2016) grouped the RCI2/PMP3 family into groups Ia (hydrophobic end), Ib (charged end), and II (hydrophilic C-terminal tail) based on hydropathy and extension of the C-terminal tail. However, a few proteins, such as SNA3/4 in yeast and CsaRCI2C in cucumber, have an N-terminal tail. These types of proteins were subdivided into PMP3-like proteins by Kwok et al. (2020).

The best known functions of the C-terminal tail in RCI2s are related to PMP3 (SNA1) complementation of NaCl sensitivity in knockout mutant yeast. Navarre and Goffeau (2000) reported that the function of RCI2s was to modulate membrane potential, which is related to NaCl sensitivity in yeast. Other reports suggested that if the RCI2s had a tail at the C-terminus, then Δ*pmp3* yeast did not recover NaCl tolerance, but C-terminal tail truncated RCI2s could recover their NaCl tolerance (Medina et al., 2007). In camelina, CsRCI2s also exhibited functional complementation of Δ*pmp3* yeast if they did not have a tail (Kim et al., 2016). However, when a C-terminal tail was added to a no-tail type, then it could not complement NaCl tolerance when expressed in Δ*pmp3*. In *Escherichia coli*, YqaE has been identified as an RCI2 homolog. Unlike yeast PMP3, mutant *E. coli* that lacked YqaE were sensitive to both deficiency and excess concentration of NaCl (Kwok et al., 2020). Interestingly, this sensitivity to NaCl deficiency in prokaryote *E. coli* was complemented by expression of other RCI2 homologs, such as MFP1a from marine dinoflagellates belong into eukaryote. Additionally, YqaE expression decreased when *E. coli* was incubated in 200 mM KCl, but yeast PMP3 and dinoflagellate MFP1a expression increased. These results suggest that RCI2s have individual response to Na^+^ and K^+^ sensitivity for each kingdom. In plant kingdom, *atrci2a* knockout mutant *Arabidopsis* showed NaCl sensitivity than wild type (WT) but recovered by over-expression of MpRCI from *Musa paradisiac*. Interestingly, MpRCI over-expressed *atrci2a* plant showed increased KCl sensitivity while *atrci2a* knockout mutant was tolerant to 150 mM KCl stress than WT (Liu et al., 2012). This suggest that RCI2 proteins played important roles in Na^+^ and K^+^ cellular ionic balance in each kingdom. Therefore, investigation of cross-linkage between RCI2s and transporter proteins related to Na^+^/K^+^ balance will be important for further studies.

The deletion of the C-terminal tail on SNA2 in yeast meant that SNA2 became localized on the PM rather than the ER (Renard et al., 2010). This suggested that the C-terminal tail might have a key destination sequence that allowed the protein to be sent to the right location. However, truncation of the RCI2 N-terminal tail was not investigated in the Δ*pmp3* complementation experiment. A future study using C-terminal tail-deleted RCI2-over-expressing plants is required if the different functions of the tail in RCI2s are to be fully understood.

## Subcellular Localization and Endocytic Trafficking of RCI2 Proteins

**FIGURE 2:**
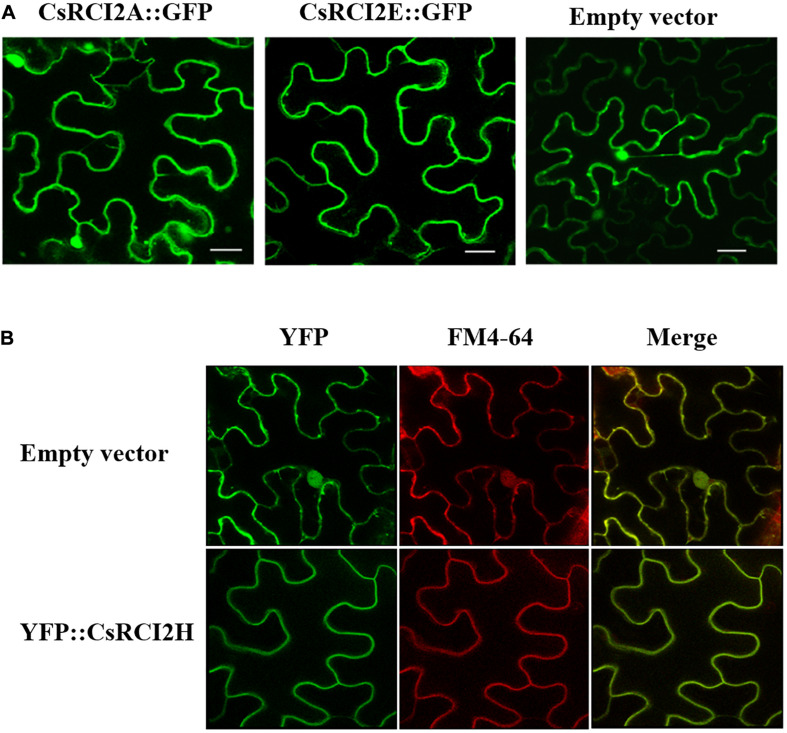
Subcellular localization of CsRCI2A/E-GFP and YFP-CsRCI2H proteins in tobacco. Confocal laser scanning microscope images of tobacco cells expressing GFP and YFP fused to CsRCI2A/E **(A)** (Kim et al., 2016) and CsRCI2H **(B)** (Kim et al., 2020) proteins, respectively. The red fluorescence signal represent FM4-64 stained lipids and PM in cell. White scale bars represent 20 μm.

Recent studies have shown that most of the RCI2 proteins are localized at the PM in *Arabidopsis*, maize, alfalfa, barrel medic, rice, and wheat (Medina et al., 2007; Rocha, 2016). In camelina, the GFP or YFP fused RCI2s, such as CsRCI2A/E/H, have been reported to localized at the PM (Figure 2; Kim et al., 2016, 2020). However, these CsRCI2s, including CsRCI2A, CsRCI2B, CsRCI2C, CsRCI2E, CsRCI2F, and CsRCI2G, were also found on endomembrane vesicles (Supplementary Videos 1–6). These RCI2s-vesicles were also observed around the PM and vacuoles in cells when transiently expressed in tobacco leaf. Furthermore, several vesicles emerged from the PM and then moved to the intracellular space, which is similar to endocytosis. However, the reason of occurrence of RCI2-vesicle trafficking has not been properly reviewed in plants. The unique two TMD structure of RCI2s has been shown to be due to an integral PM protein. Therefore, methods in previous studies involved fusion of LTI6a (AtRCI2A) and LTI6b (AtRCI2B) with non-polar PM marker proteins that could be used as tracers in high-resolution live imaging systems (Grebe et al., 2003; Maizel et al., 2011; Hosy et al., 2015). This tracer system has been used to investigate RCI2-associated endocytic trafficking in studies pertaining to the endocytosis of transporter proteins (Table 1). The RCI2s are considered to be PM proteins bound to phospholipid, and GFP-fused LTI6a and LTI6b-over-expressing *Arabidopsis* plants have helped to elucidate endocytic trafficking of PIN2 and aquaporins (Kleine-Vehn et al., 2011; Li et al., 2011; Wudick et al., 2015). When the LTI6s were treated with brefeldin A [BFA, aggregation reagent for endocytic vesicles trapped in the trans-Golgi network and early endosomes (EEs)], the results showed that they were localized to the early endosomes (EEs). When *Arabidopsis* roots were treated with cycloheximide (CHX) for 15 and 30 min to limit protein synthesis, the Lti6a-GFP protein clearly internalized to BFA bodies (Wang C. et al., 2013). Also, GFP-LTI6b expression was observed at the vacuole in *Arabidopsis* under NaCl stress (Ueda et al., 2016). Yeast that lacked the PMP3 protein showed delayed endocytosis of lipid-specific dye FM4-64 in their cells. Furthermore, Δ*PMP3* yeast showed decreased expression of membrane proteins and increased expression of endocytosis-related proteins in Δ*PMP3* compared to the WT (De Block et al., 2015). These results suggested that RCI2s could be localized at the PM, EEs, or vacuoles through the endocytic vesicle trafficking pathway.

**TABLE 1:** Subcellular localization or endomembrane trafficking of RCI2/PMP3 proteins established by recent studies.

Name	Species	Accession number	Protein interaction	Sterol co-localization	TyrA23 sensitivity	Experiment type	Characteristics	References
AtRCI2B (LTI6b)	*A. thalina*	At3g05890	ND	ND	YES	GFP-fusion, confocal laser microscope, FRAP	Lti6b BFA bodies do not accumulate after TyrA23 treatment. Lti6b can recover after photobleaching with or without TyrA23 treatment.	Dhonukshe et al. (2007)
AtRCI2A (LTI6a)	*A. thalina*	At3g05880	ND	YES	ND	GFP-fusion, confocal laser microscope	EGFP-Lti6a co-localized at BFA body enriched sterols stained by filipin	Grebe et al. (2003)
AtRCI2B (LTI6b)	*A. thalina*	At3g05890	ND	ND	ND	GFP-fusion, FRAP	Non-polar PM marker rapid recovery after photobleaching compared to PINs	Kleine-Vehn et al. (2011)
AtRCI2A (LTI6a)	*A. thalina*	At3g05880	ND	ND	ND	GFP-fusion, confocal laser microscope	Discontinuous labeling by GFP-PIP2;1 but LTI6a labled entire PM. GFP-LTi6a displayed strikingly faster lateral diffusion than GFP-PIP2;1	Li et al. (2011)
AtRCI2B (LTI6b)	*A. thalina*	At3g05890	ND	ND	ND	High−resolution live imaging	Membrane lavel protein for live cell imaging	Maizel et al. (2011)
AtRCI2B (LTI6b)	*A. thalina*	At3g05890	ND	ND	ND	Reconstruction of ProUBQ10:LTI6b-mEOS, Lateral diffusion and local density analysis	Higher local density of fluorescent particles. Insensitive to a sorbitol treatment and does not show higher clustering than PIP2;1 under hyperosmotic stress	Martiniere et al. (2019)
AtRCI2A	*A. thalina*	At3g05880	ND	ND	ND	GFP-fusion, confocal laser microscope	Auxin inhibits GFP-RCI2A endocytosis	Pan et al. (2009)
AtRCI2B (LTI6b)	*A. thalina*	At3g05890	ND	ND	ND	GFP-fusion, confocal laser microscope	Internalized into vacuole lumen under 100 mM NaCl for 6 h	Ueda et al. (2016)
CsRCI2A	*C. sativa*	XR_002036656.1	ND	ND	ND	GFP-fusion, confocal laser microscope	Localized at PM and intracellular vesicles	Kim et al. (2016)
CsRCI2E	*C. sativa*	XM_019231129.1	CsPIP2;1	ND	ND	Co-IP, *X. laevis* swelling assay	Interacts with CsPIP2;1 to reduce water transport	Kim et al. (2019)
CsRCI2F	*C. sativa*	XM_010434658.2	CsPIP2;1	ND	ND	Co-IP, *X. laevis* swelling assay	Interacts with CsPIP2;1 to reduce water transport	Kim et al. (2019)
PMP3 (SNA1)	*S. cerevisiae*	SGD:S000002684	ND	ND	ND	GFP-fusion, confocal laser microscope	Endocytosed into the vacuole. Induces endocytosis related PM proteins in Δpmp3	De Block et al. (2015)
SNA3	*S. cerevisiae*	SGD:S000003687	ND	ND	ND	GFP-fusion, confocal laser microscope	RSP5 ubiquitination regulates sorting of SNA3 in the MBV pathway	MacDonald et al. (2012)
SNA2	*S. cerevisiae*	SGD:S000007236	ND	ND	ND	GFP-fusion, confocal laser microscope	Two YXXQ motifs affect relocalization of SNA2p	Renard et al. (2010)

*ND, not determined.*

Endocytosis is a basic metabolism in living organism that participating remove, recycle, and degrade of proteins from PM (Rodriguez-Furlan et al., 2019). It maintains cell homeostasis by fine-tuning the PM system to adapt to biotic and abiotic stresses in plants (Wang et al., 2020). Internalization of major PM transporter compounds, such as water transporter aquaporins and auxin transporter PIN2, has been reported to regulate water homeostasis and cell development, respectively (Kleine-Vehn et al., 2011; Li et al., 2011; Luu et al., 2012). The endocytosis of PIP2s has been shown to be internalized through both clathrin-mediated endocytosis (CME) and the sterol-raft system under severe osmotic stress, including NaCl shock (Li et al., 2011; Pou et al., 2016). However, it has also been reported that the PIN2 pathway is only regulated by CME as evidenced by a pharmacological test using tyrphostin A23 (TyrA23), which inhibits CME (Dhonukshe et al., 2007). Interestingly, a study on PIP2 and PIN2 endocytosis also tested LTI6b as a reference PM protein (Wang C. et al., 2013). In their results, expression of GFP-LTI6b revealed endocytic trafficking and CME features after treatment with BFA. These results suggested that RCI2 proteins could be internalized into the endomembranes. Additionally, the accumulation of LTI6a and LTI6b BFA bodies was inhibited by treatment with auxin and TyrA23 (Dhonukshe et al., 2007; Pan et al., 2009). This implied that LTI6a and LTI6b-associated endocytosis was regulated by hormone signals and CME. The RCI2/PMP3 dynamics were confirmed by other studies when the LTI6s were introduced as PM marker proteins. The density results for GFP-LTI6b fluorescence protein particles after they had been recovered following photo bleaching showed that they had a higher velocity than that of GFP-AtPIP2;1 (Dhonukshe et al., 2007). Furthermore, LTI6b had a greater rapid diffusion co-efficiency compare than other PM proteins, such as PIP2;1 and AHA2, when a FRAP (fluorescence recovery after photobleaching) test was carried out (Martiniere et al., 2019). Additionally, the velocity of LTI6b re-localization was faster than that for PIP2;1 and PIN2, but slower than total lipid stained by FM4-64 (Boutté et al., 2010). This information suggests that the RCI2 proteins have characteristics that would support rapid trafficking. However, LTI6a/b are typical no-tail type RCI2 proteins, which suggests that future studies should characterize tail-type RCI2 proteins. The function of tail-type RCI2s is not well-established in plant species, but significant results have been revealed by several reports on yeast. In yeast, four PMP3 protein families can be divided into a no-tail (PMP3/SNA1) family and tail type families (SNA2, SNA3, and SNA4). Deletion of PMP3 increases NaCl sensitivity, but it is reduced by RCI2 homologs that have no tails. PMP3 has been considered to be a membrane potential regulator because Na^+^ uptake increased when PMP3 was deleted (Nylander et al., 2001). De Block et al. (2015) reported the role played by PMP3 in yeast PM proteins. They found that yeast that lacked PMP3 had an altered PM protein composition that led to increased amounts of endocytosis-related proteins compared to the WT. They also reported that the Δ*pmp3* yeast showed delayed endocytosis of FM4-64 stained vesicles compared to the WT, and that PMP3 protein could bind to phosphorylated phosphoinositides, phosphatidic acid, and sphingomyelin. The growth of Δ*pmp3* yeast was affected because it could not metabolize sphingolipid. These results suggest that PMP3 plays an important role in the endocytic trafficking pathway and that it interacts with inositides and sphingolipid.

Yeast SNA2 is a tail type RCI2/PMP3 protein that has a hydrophilic tail in the C-terminal region. Renard et al. (2010) reported that point mutation of the YXXQ motif in C-terminal residues changed the destination of SNA2 protein during the re-localization process. Furthermore, their report suggested that two tyrosine-based sorting motifs in SNA2 played a key role in the localization pathway. SNA3 belongs to the PMP3-like proteins, which have relatively long C-terminal tails compared to other tail type RCI2s. SNA3 has been reported to interact with RSP5, a major ligase responsible for ubiquitination of proteins (Stawiecka-Mirota et al., 2007; Lauwers et al., 2010; MacDonald et al., 2012). It is an adapter protein for RSP5 which acts via the PY (PPXY) motif in the C-terminal residue and is sorted in multiple vesicular bodies. Additionally, it has been found that SNA3 protein lacks the lysine residues found in the cytosolic region of K19 (N-terminal) and K125 (C-terminal), which affects SNA3 protein trafficking to the vacuole (MacDonald et al., 2012). These results indicated that the PMP3-like structure might be involved in specialist protein binding and could induce endomembrane sorting. Furthermore, reports have suggested that the C-terminal peptide properties of RCI2/PMP3 may contribute to the final destination of vesicles containing RCI2/PMP3.

## Function of RCI2-Induced Vesicle Trafficking

Previous reports have suggested that NaCl or osmotic stress induces endocytosis of PIP2s (Li et al., 2011; Chevalier and Chaumont, 2015; Pou et al., 2016). Under hyperosmotic conditions, PIP2s are internalized into the intracellular region by CME or sterol-raft induced endocytosis. These mechanisms are known to maintain water homeostasis under osmotic stress. The PIP2s contribute to water transportation into cells, and they are also involved in the removal of compounds from cells, which is based on the outside osmolarity of the cell. The water transport activities of PIPs are regulated by phosphorylation, voltage-sense gating, and internalization into cells. Additionally, the Na^+^ transport activity of AtPIP2;1 is regulated by its interaction with AtPIP1;2 (Byrt et al., 2017). The PIP2;1 channel ion transport pathway needs to be inactivated if cell homeostasis is to be maintained under serious NaCl stress. Internalized PIP2s are sorted in vesicles and vacuoles for recycling or degradation (Ueda et al., 2016). The protein interactions between tail-type CsRCI2E/F and CsPIP2;1 have been analyzed using co-immunoprecipitation and a *Xenopus laevis* oocyte swelling assay in camelina (Kim et al., 2019). Purified PM protein pull-down assay using CsRCI2E and CsRCI2F antibodies showed a CsPIP2s band after immunoblotting with PIP2s antibodies. Expression of CsRCI2E and CsRCI2F reduced the water transport activity of CsPIP2;1 when co-expressed in the *X. laevis* oocyte. Furthermore, CsPIP2;1 abundance at the oocyte membrane decreased after co-expression with CsRCI2E and CsRCI2F. This suggests that deactivation of CsPIP2;1 by internalization can reduce Na^+^ influxes into cells under NaCl stress.

The H^+^-ATPase in the PM is a key regulator of NaCl tolerance because it generates a proton driving force for Na^+^/H^+^ exchange by SOS1 (Shi et al., 2000; Zhu, 2003). Moreover, increased PM H^+^-ATPase activity has also been reported to contribute to cold stress tolerance in many plant species (Janicka-Russak et al., 2012). In *Arabidopsis atrci2a* mutant complemented by MpRCI2 over-expression, the PM H^+^-ATPase activity was increased under NaCl stress than WT but the function for RCI2-induced H^+^-ATPase activation was not concluded (Liu et al., 2012). The activation of PM H^+^-ATPase regulated by several conditions such as phosphorylation of penultimate C-terminal threonine residue, interaction with 14-3-3 proteins, and abundance of phosphatase PP2C in cytosol (Spartz et al., 2014). Moreover, hormonal endocytic trafficking of PM H^+^-ATPase have been reported by Xia et al. (2019). In the result, SYP132-induced endocytic trafficking of PM H^+^-ATPase from PM was inhibited by auxin, but promoted by ABA (abscisic acid). Meanwhile, auxin treatment causes inhibition of RCI2s endocytosis but ABA treatment can induce RCI2 expression (Medina et al., 2007; Pan et al., 2009). It means that both auxin and ABA can affect PM H^+^-ATPase activity and/or endocytosis of RCI2s. However, the relationship between RCI2 and PM H^+^-ATPase requires additional analysis of the RC12 type when it is subjected to various stress conditions. Also, status of RCI2s and PM H^+^-ATPase trafficking should be considered in further study.

The expression of most RCI2 proteins are rapidly induced by abiotic stresses, and high RCI2 expression levels under abiotic stresses have been confirmed by many reports (Nylander et al., 2001; Medina et al., 2007; Kim et al., 2016; Rocha, 2016). It has been suggested that RCI2 regulates membrane potential under cold, drought, and salt stress (Kim et al., 2016; Kwok et al., 2020). To date, other independent functions related to RCI2 proteins have not been adequately investigated, but their characteristics imply that they can be used to explore cell functioning. Therefore, they are often used as a PM reference protein during live cell imaging when fused with fluorescence proteins (Li et al., 2011). Many reports have indicated that RCI2 proteins can travel from the PM to the ER or EE via the endocytic trafficking pathway. It has also been shown that RCI2B (Lti6b) treated with BFA can mediate the localization of RCI2B at BFA bodies, but it has been reported to be inhibited by TyrA23 (Dhonukshe et al., 2007). This result indicated that endocytic trafficking of RCI2A was part of the CME pathway because TyrA23 is a known inhibitor of CME. In addition, Wang C. et al. (2013) concluded that endocytosis of RCI2A-GFP is clathrin-dependent because the numbers of RCI2A-GFP labeled BFA bodies were changed in clathrin light chain double knockout (*clc2-1* and *clc3-1*) *Arabidopsis*. However, EGFP-RCI2B was observed in the BFA body along with sterols stained by the sterol-binding dye filipin (Grebe et al., 2003). Plant cells have a sterol-raft endocytic pathway and AtPIP2;1 has been reported to be internalized by both the CME and the sterol-raft pathway (Li et al., 2011). Interestingly, PIP2;1 has been shown to interact with CsRCI2E and CsRCI2F in camelina (Kim et al., 2019). This suggested that RCI2 might be related to the sterol-raft pathway. The evidence suggests that RCI2s are involved in both the CME and Sterol-raft pathways, but only results for a few of RCI2A and RCI2B are currently reported. Therefore, focusing on endocytic trafficking of unlabeled RCI2 members related to CME or sterol-raft pathway are required for further study.

## Concluding Remarks

Many plant species have RCI2 family (Rocha, 2016). Unlike animal, survival of plants depends on its natural born stress tolerance or dynamic adaptation from various environmental conditions. The RCI2s exhibit dynamic expression, cellular diffusion, and endocytic trafficking (Medina et al., 2007; Kim et al., 2019; Martiniere et al., 2019). Most of RCI2s studies concluded that RCI2s participate wide range of stress tolerance (Liu et al., 2012; Kim et al., 2016, 2020). However, the published studies of RCI2s in plants did not make a linkage with cellular functions despite the several RCI2s were already been used to tracking membrane proteins redistribution through live cell imaging (Li et al., 2011; Maizel et al., 2011; Hosy et al., 2015). Moreover, recent reports of RCI2s live cell imaging was carried out through pharmacology process such as FM4-64 and NaCl even the RCI2s have individual characteristics to temperature stress (Ueda et al., 2016; Kim et al., 2020). Therefore, exploring RCI2s dynamics under various stresses such as cold and heat might be provided novel mechanisms of RCI2-dependent stress tolerances.

The RCI2s have unique structure containing two TMD and hydrophilic C-terminal tail and can divide into subgroups by composition of N- or C-terminal extension (Kwok et al., 2020). C-terminus studies of SNA2 and SNA3 in yeast revealed that RCI2s have motifs to binding other proteins or molecules which is guide to final destination (Stawiecka-Mirota et al., 2007; Renard et al., 2010; MacDonald et al., 2012). Also, CsRCI2E and CsRCI2F in camelina as a tail type showed protein interaction with PIP2 which is participate PM abundance of PIP2 (Kim et al., 2019). From these evidences, C-terminus of RCI2s might have functional motifs for cellular redistribution. However, no-tail type RCI2s can travel through endocytic pathway, especially in RCI2A and RCI2B (Pan et al., 2009; Li et al., 2011; Maizel et al., 2011; Ueda et al., 2016). These evidences make question to possible binding affinity from two TMD structure. Therefore, characterization of motif-specific RCI2s traffic regulation might be a tool for regulation of membrane proteins distribution for crop of the desired form.

**FIGURE 3:**
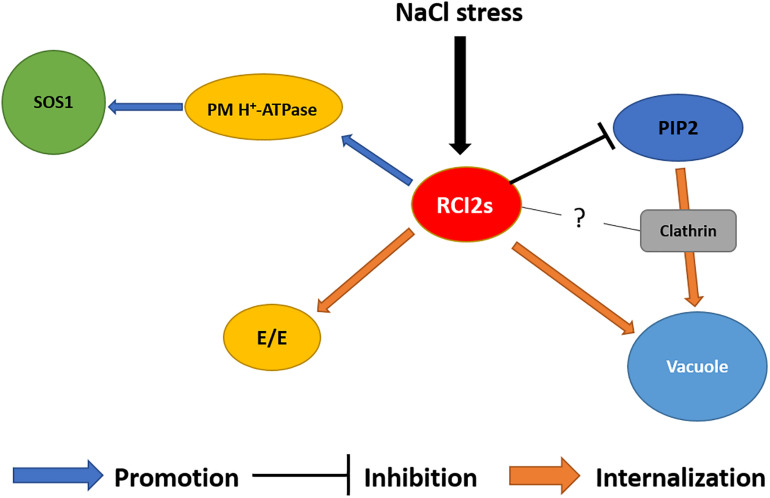
Schematic diagram of the RCI2/PMP3-related NaCl stress tolerance mechanism. Expression of RCI2s promotes PM H^+^-ATPase activity to generate a proton driving force for the Na^+^/H^+^ exchanger SOS1. The NaCl-induced RCI2s inhibit water transport activity of aquaporin PIP2, which transports Na^+^ into the cell. Under NaCl stress, PIP2s were internalized into the vacuole via CME. Subcellular localization of RCI2s was observed in the PM and endomembranes, including early endosomes (EEs) and the vacuole. The NaCl-induced RCI2s contribute to the NaCl stress defense mechanism by regulating the activity and reorganization of PM proteins, which helps maintain ion homeostasis in the plant cell.

In conclusion, the results indicated that the expression of RCI2s proteins was related to internalization of PM proteins, and that the regulation of major transporter protein activity altered the strategy used to maintain cell homeostasis under abiotic stresses (Figure 3). However, a number of aspects about the RCI2 properties need to be investigated. The aspects are: (i) does RCI2 protein directly mediate vesicle formation? (ii) What is the function of RCI2 under temperature stress; and (iii) which PM protein binding motifs regulate RCI2 binding? Furthermore, internalization of RCI2 has not been fully elucidated in relation with CME or sterol-raft endocytosis, and has not been investigated for all subgroups of RCI2s. The expressions of the *RCI2* genes showed that their individual transcriptions were affected by the type, intensity, and duration of the abiotic stresses. Therefore, focusing on the individual roles of RCI2 members under abiotic stress will improve our understanding of the internalization of PM proteins, their effects on cell homeostasis, and the mechanisms that help cells adapt to adverse conditions.

## Author Contributions

H-SK and WP contributed to the review conception, design, and writing of the manuscript. H-SL and J-HS assisted information and data arrange from literature. S-JA supervised the entire study including manuscript editing. All authors contributed to the completed manuscript.

## Conflict of Interest

The authors declare that the research was conducted in the absence of any commercial or financial relationships that could be construed as a potential conflict of interest.
