# Supplementary material for: Subcellular Journey of Rare Cold Inducible 2 Protein in Plant Under Stressful Condition
Source: Front Plant Sci. 2021 Jan 12;11:610251. doi: 10.3389/fpls.2020.610251 (PMC7835403; doi:10.3389/fpls.2020.610251)
Supplement: Supplementary file 1 [file Data_Sheet_1.docx]

(A)

(B)
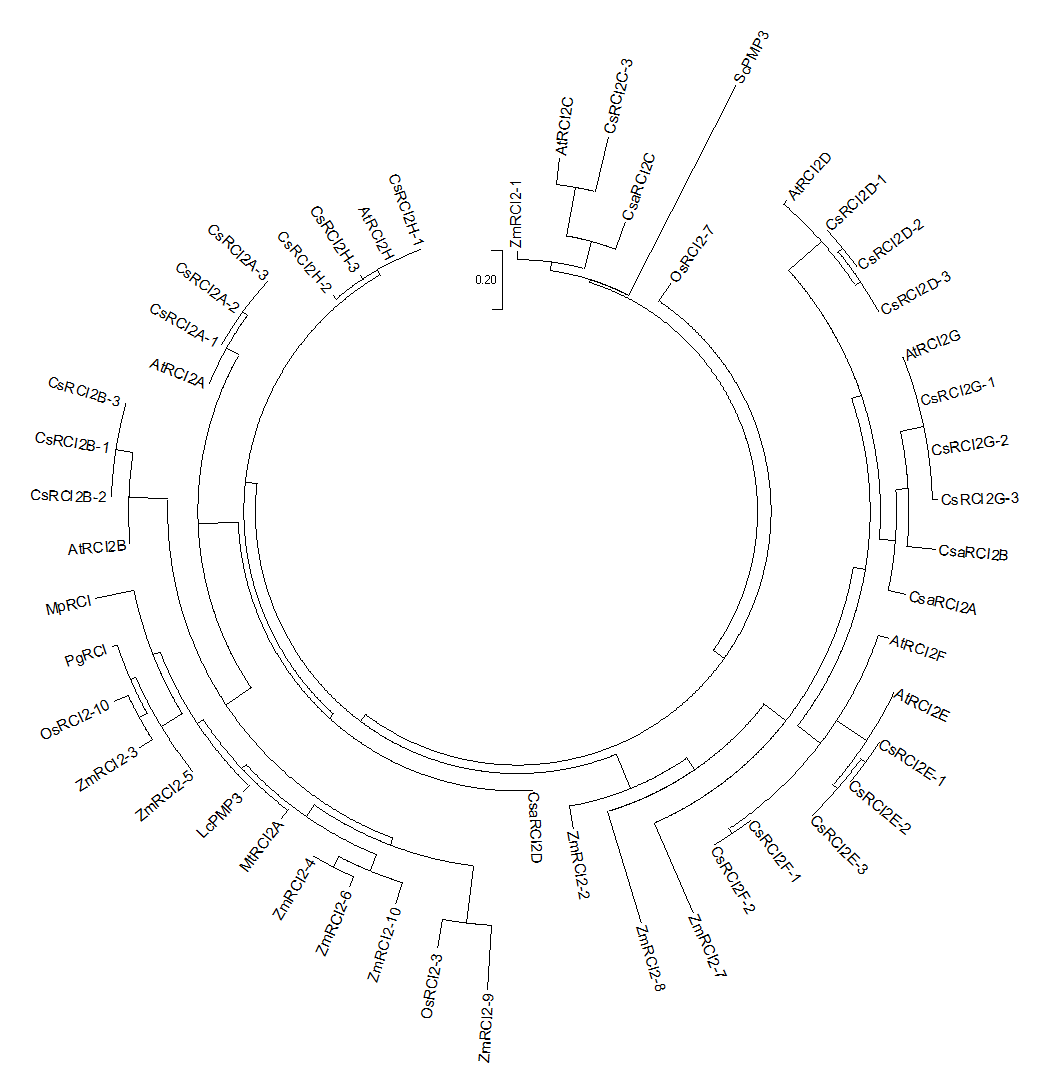


**Figure S1.** Amino acid sequence alignments of RCI2/PMP3 proteins in various plant species. (A) Sequence alignments were conducted by the CLC genomic work bench program. (B) The transmembrane domain (TMD) and the C-terminal tail region are shown on top of the amino acid sequences. The phylogenetic tree was drawn using the MEGA 7.0 program and the neighbor-joining method with 1000 replicates. The GenBank accession numbers are: camelina (CsRCI2A, JQ809231.1; CsRCI2B, XM_019236439.1; CsRCI2E, HM641262; and CsRCI2F), *Arabidopsis* (AtRCI2A to AtRCI2H; AT3G05880; AT3G05890; AT1G57550; AT2G24040; AT4G30650; AT4G30660; AT4G28088; and AT2G38905), rice (OsLTI6a, XR_003243514.1; OsRCI2-1, CP018157.1; OsRCI2-2, AP014957.1; OsRCI2-12, AP014965.1), maize (ZmRCI2-1, NM_001363954.1; ZmRCI2-2, NM_001320456.1; ZmRCI2-3, XM_020548632.3; ZmRCI2-4, NM_001158368.2; ZmRCI2-5, NM_001153931.3; ZmRCI2-6, NM_001154036.2; ZmRCI2-7 XM_035962294.1; ZmRCI2-8, NM_001158450.1; ZmRCI2-9, XM_020544370.3; ZmRCI2-10, NM_001114162.2), *Leymus chinensis* (LcPMP3, AB161676.1), *Medicago truncatula* (MtRCI2A, XM_003626084.3), *Musa paradisiac* (MPRCI, EU490796.1), cucumber (CsaRCI2A, XM_004146625.3; CsaRCI2B, XM_011650594.2; CsaRCI2C, XM_004137759.3; CsaRCI2D, XM_004143561.3), and yeast (ScPMP3p, 6320482).

(A)


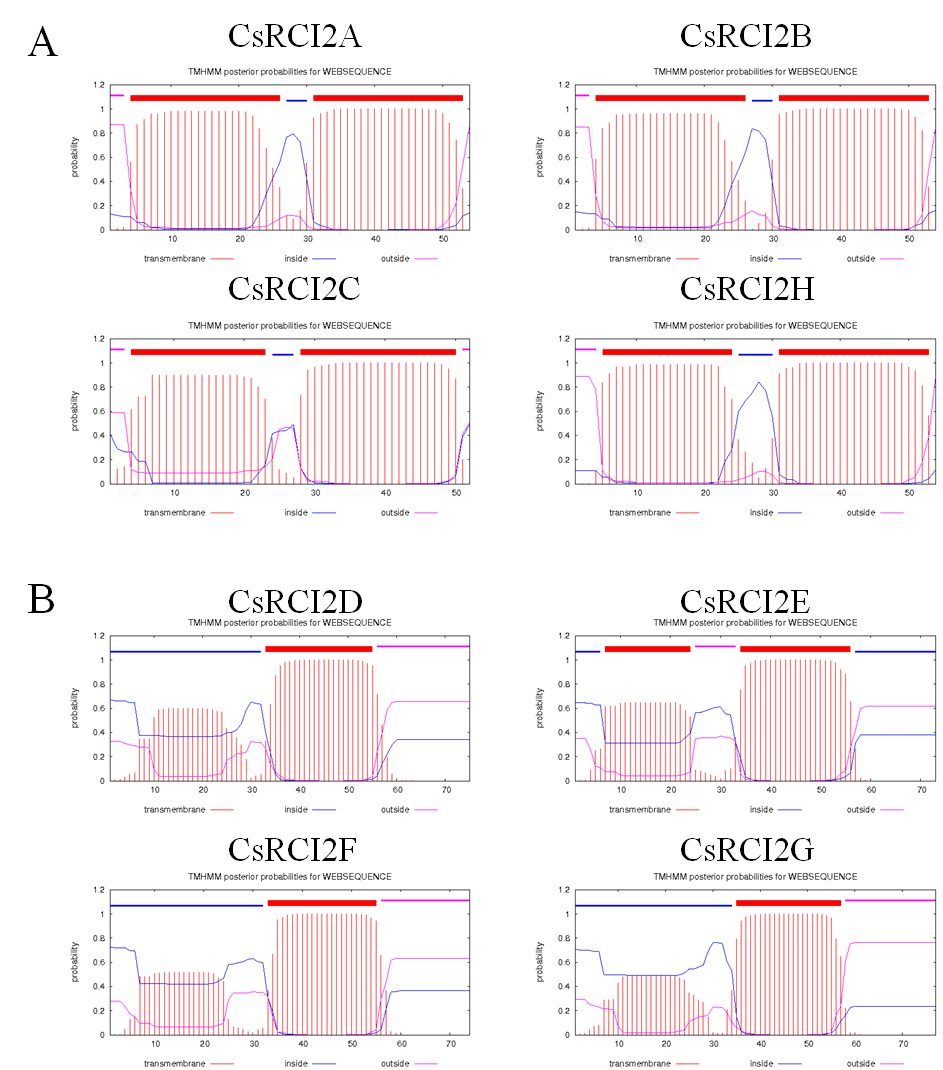


(B)


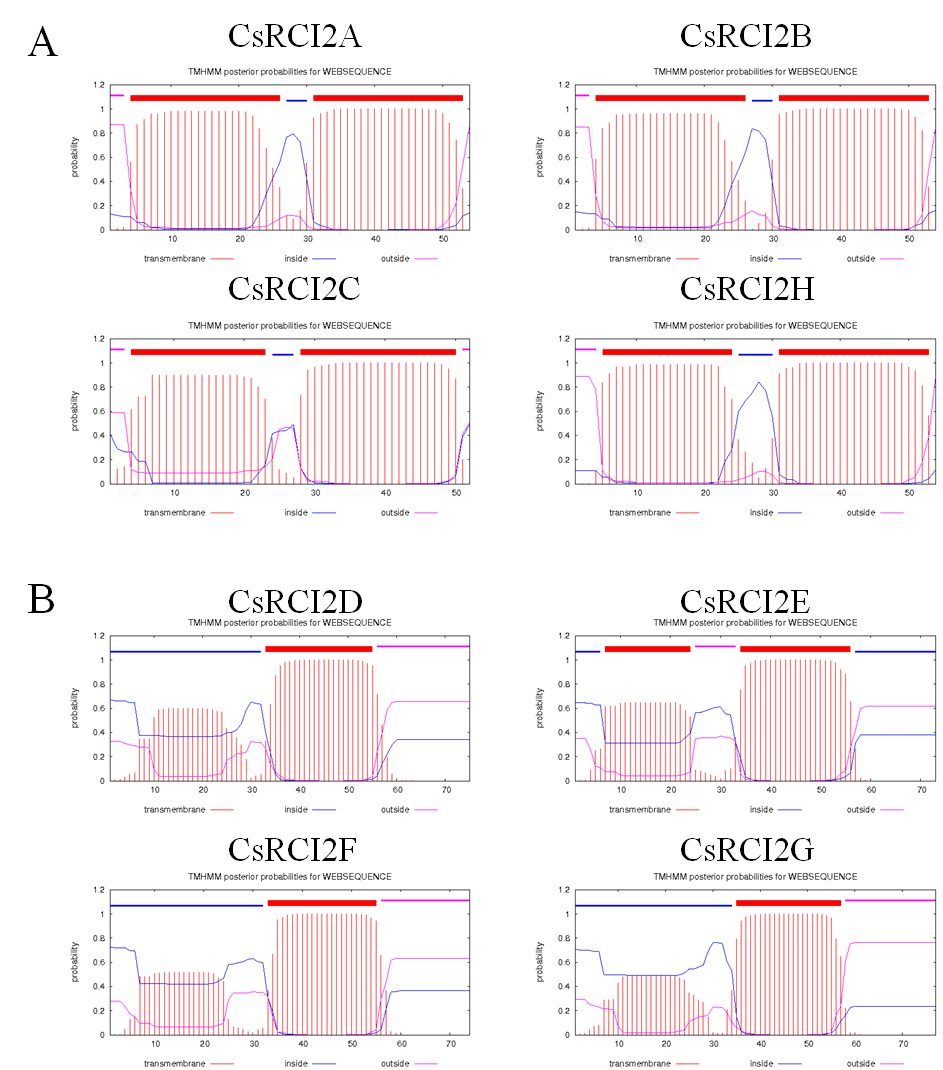


(C)


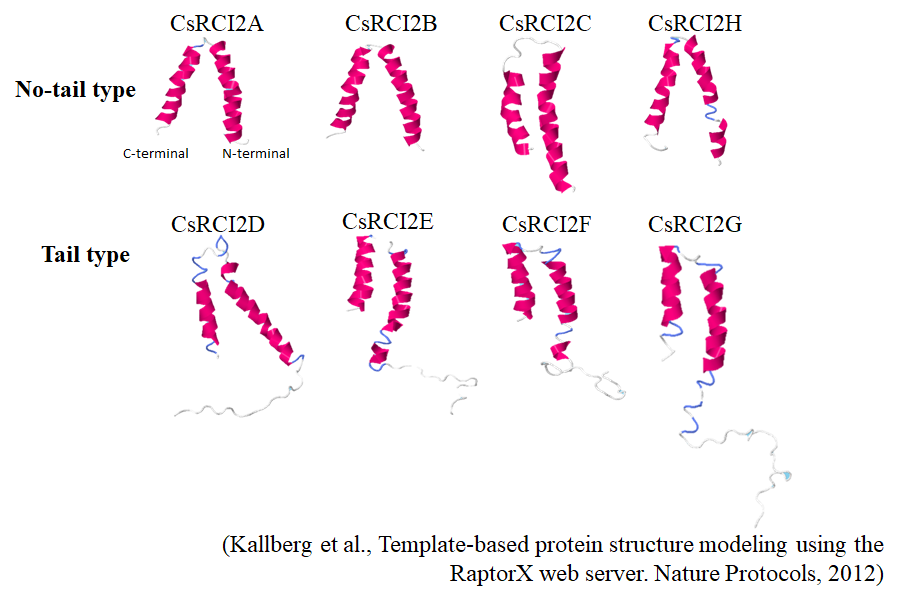


**Figure S2.** Transmembrane domain predictions of the protein sequences in CsRCI2s. Hydropathies of (A) no-tail type CsRCI2A, CsRCI2B, CsRCI2C, and CsRCI2H, and (B) tail type CsRCI2D, CsRCI2E, CSRCI2F, and CsRCI2G were analyzed by the HMTMM V2.0 server. The properties of the protein sequences are represented by the TMD region (red bar) and the direction of the protein tail inside and outside the molecule (blue bar; inside, pink bar; outside). The predicted protein structure of CsRCI2A to H were analyzed by RapterX server (http://raptorx.uchicago.edu/).
