# Supplementary material for: Subcellular Journey of Rare Cold Inducible 2 Protein in Plant Under Stressful Condition
Source: Front Plant Sci. 2021 Jan 12;11:610251. doi: 10.3389/fpls.2020.610251 (PMC7835403; doi:10.3389/fpls.2020.610251)
Supplement: Supplementary file 2 [file Table_1.DOCX]

Table S1. Gene information about the different *CsRCI2*s

| Gene name | Accession | Homolog | Homology (%) |
| --- | --- | --- | --- |
| *CsRCI2A* | JQ809231.1 | *AtRCI2A* (At3g05880) | 94.56 |
| *CsRCI2B* | XM_019236439.1 | *AtRCI2B* (At3g05890) | 92.12 |
| *CsRCI2C* | XM_019227642.1 | *AtRCI2C* (At1g57550) | 75.47 |
| *CsRCI2D* | XM_010431055.2 | *AtRCI2D* (At2g24040) | 96.05 |
| *CsRCI2E* | HM641262 | *AtRCI2E* (At4g30650) | 91.89 |
| *CsRCI2F* | XM_010434658.2 | *AtRCI2F* (At4g30660) | 90.22 |
| *CsRCI2G* | XM_019233932.1 | *AtRCI2G* (At4g28088) | 100.00 |
| *CsRCI2H* | XM_019245705.1 | *AtRCI2H* (At2g38905) | 100.00 |
